# Parenthood and care in academia: Publication counts, self-assessed productivity, and gendered differences

**DOI:** 10.1371/journal.pone.0355683

**Published:** 2026-09-16

**Authors:** Matteo A. Ruberto, Ludovica Alesci, Salvatore Maione, Valentina Rotondi

**Affiliations:** 1 Department of Business Economics, Health and Social Care (DEASS), University of Applied Sciences and Arts of Southern Switzerland (SUPSI), Manno, Switzerland; 2 Nuffield College and Leverhulme Centre for Demographic Science, University of Oxford, Oxford, United Kingdom; Institut National d'Etudes Demograpiques: INED, FRANCE

## Abstract

How does parenthood relate to academic productivity when we look beyond publication counts? We synthesize evidence from five small-scale meta-analyses and present original survey data from academic professionals combining (i) objective outcomes (publications per year and a standardized real-effort task), (ii) subjective assessments (self-reported productivity loss), and (iii) qualitative evidence from open-ended responses on perceived (dis)advantage and work identity. Meta-analytic estimates are statistically indistinguishable from zero and characterized by extreme heterogeneity, suggesting limits of publication-based metrics for capturing care-related mechanisms. In our survey, parenthood is not robustly associated with objective productivity once demographic, occupational, and caregiving covariates are considered, including in within-individual panel fixed-effects models. By contrast, perceived productivity loss rises nonlinearly with within-couple childcare imbalance, with stronger effects when respondents shoulder a dominant share of care and clear gender heterogeneity. Multinomial models and text analysis show a lexical and evaluative divergence: academics who reject parenthood as an advantage emphasize time scarcity and care responsibilities, while those affirming an advantage invoke focus, efficiency, and personal development. We argue that the apparent inconsistency of parenthood effects reflects a mismatch between *what* productivity metrics count and *how* caregiving reshapes constraints, capacities, and evaluation. We discuss design principles for academic assessment that better align recognition with performance under care constraints.

## Introduction

A large literature on parenthood has examined labor force participation and wages; more recently, attention has shifted to *productivity* [1]. Unlike employment or earnings, productivity is multidimensional and context-dependent: it may capture output volume, task efficiency, or work quality, and it varies across occupations and institutional settings. As a result, empirical studies often default to narrow observable indicators that may overlook dimensions of work shaped by caregiving.

A central challenge is therefore *measurement*: standardized indicators of productivity are scarce and frequently misaligned with actual work processes [2]. In addition, selection into continued employment after childbirth—especially for mothers—can bias comparisons [3], while organizational responses to parenthood (e.g., task reassignment or availability heuristics) can affect measured performance independently of underlying ability [4]. These features suggest that inconsistent findings in the literature may reflect the limits of prevailing metrics rather than the absence of meaningful effects. Such misalignment has broader equity implications: evaluation systems that rely on narrow output metrics risk undermining SDG 4 and SDG 5 by under-recognizing performance produced under unequal care constraints.

Academia offers a revealing setting to investigate these issues. On the one hand, performance indicators are easily identifiable and public (e.g., publication counts, citations, grants); on the other, academic careers are high-pressure, loosely structured, and cumulatively evaluated, making measurement choices especially consequential. Heterogeneity in leave policies, childcare support, and workload allocation further sharpens the interplay between caregiving and evaluation. Parental penalties in academic productivity, particularly among women, may arise through multiple interconnected mechanisms, including increased time constraints due to caregiving responsibilities, cumulative career disadvantages over time, evaluation biases and ‘maternal wall’ stereotypes in academic environments, as well as unequal divisions of care within couples and the role played by partners in supporting or constraining academic work. Against this backdrop, studies document divergent patterns. Early evidence showed lower publication output among women with young children, plausibly mediated by reduced collaboration [5]. More recent work points to mixed effects, suggesting that declines in productivity are more likely to emerge when parents are heavily engaged in caregiving activities associated with parenthood—particularly when caregiving demands are intensive—rather than from the transition to parental status itself [6]; short-term increases around childbirth followed by slowdowns, particularly for mothers, alongside slower growth in visibility [7]. Other contributions emphasize a *fatherhood premium*, with parents (especially fathers) outperforming non-parents in some contexts and gaps attenuating where supportive policies are strong [8,9]. Institutional regimes and gender norms around leave and care shape continuity and advancement [10,11], with substantial disciplinary heterogeneity and evidence of perceived prejudice toward academic mothers [12–14]. Taken together, the literature does not identify a single, generalizable effect of parenthood on academic productivity. Instead, findings vary by context, discipline, career stage, and caregiving arrangements; however, the literature more consistently documents penalties for mothers, whereas fathers often experience no comparable disadvantage and may even benefit from a fatherhood premium.

We argue that this inconsistency reflects not only genuine heterogeneity but also a deeper conceptual issue: a systematic mismatch between *what* academic productivity metrics count and *how* caregiving reshapes constraints, capacities, and contributions. We advance the literature in three ways. First, we provide five targeted small-scale meta-analyses comparing women and men, parents and non-parents, mothers and non-mothers, fathers and non-fathers, and mothers and fathers, using them diagnostically to characterize heterogeneity and the limits of publication-based metrics. Second, we present original survey evidence from two samples of academic professionals, combining two objective measures of productivity—bibliometric indicators and a standardized real-effort task—with a subjective measure based on self-reported productivity. This approach allows us to compare observable academic output, performance under constrained conditions, and perceived effectiveness within a unified evaluative framework. Third, we integrate qualitative evidence from open-ended responses to examine how academics interpret the work–care interface, including self- and colleague-related perceptions.

Our results show no robust association between parenthood and objective productivity (publications per year; task performance), but a strong and non-linear rise in *perceived* productivity loss as caregiving intensity and imbalance increase, with clear gender heterogeneity. This divergence suggests a structural gap between how productivity is measured and how it is lived. By reframing parenthood as a stress test of prevailing evaluation practices, we contribute to debates on gender inequality, performance assessment, and institutional design in academia. Recognizing the limits of output-based measures is essential for building more equitable and accurate systems of academic evaluation that acknowledge not only visible achievements but also the hidden value and constraints generated by care. Rather than asking whether parenthood lowers academic productivity, our findings suggest that publication-based metrics alone may have limited ability to fully capture the relationship between parenthood and academic productivity, particularly when productivity may also be shaped by less tangible factors such as caregiving responsibilities, changing priorities, and evaluation processes.

## Results

### Meta-analytic evidence

To evaluate whether the existing literature converges on systematic differences in academic publication output by gender and parental status, we conducted a series of small-scale meta-analyses.

The relatively small number of studies included in each meta-analysis does not reflect a deliberate preference for small-scale synthesis, but rather the limited availability of studies meeting strict inclusion criteria. In particular, restricting attention to comparable outcomes, consistent measurement, and sufficiently homogeneous empirical designs substantially reduces the number of eligible estimates.

Expanding the pool would have required relaxing these criteria, at the cost of introducing substantial heterogeneity in constructs and measurement. In this sense, we prioritize internal comparability over breadth, and interpret the resulting analysis as a focused quantitative synthesis rather than a comprehensive aggregation of the literature.

While this limitation is non-trivial and restricts external validity, we believe it is preferable to maintain strict comparability rather than inflate the sample at the cost of conceptual inconsistency.

Therefore, rather than estimating a single average effect, the goal is diagnostic: to assess whether publication-based measures of academic productivity yield consistent conclusions across studies and contexts. Detailed effect size estimates, confidence intervals, heterogeneity statistics, and tests for publication bias are reported in the Supplementary Materials.

Across all comparisons, pooled effect sizes are statistically indistinguishable from zero and marked by exceptionally high heterogeneity. This combination does not imply the absence of meaningful relationships; instead, it signals substantial contextual variation and limited convergence among studies relying on publication-based indicators. The key insight is not simply that estimates are heterogeneous, but that heterogeneity is so extreme that pooling becomes substantively uninformative. This is itself an empirical finding about the limits of publication-based productivity metrics. Importantly, following the suggestion of an anonymous reviewer, we assessed the robustness of our findings by excluding the study conducted within the Russian academic system, given its potentially distinct institutional and tenure structure relative to the other countries included in the sample [15,16]. The results remain highly robust to this exclusion: neither the statistical significance nor the substantive interpretation of the estimated effects changes, confirming that the overall findings are not driven by the specific characteristics of the Russian academic context.

The Supplementary Materials summarize the results for five comparisons (women versus men, parents versus non-parents, mothers versus childless women, fathers versus childless men, and mothers versus fathers). In every case, confidence intervals are wide and overlap zero, while between-study heterogeneity is extreme (*I*^2^ > 99%). Importantly, this lack of convergence persists even when tests detect no publication bias.

Taken together, the meta-analytic evidence highlights a central limitation of the literature: differences in academic productivity associated with gender and parenthood cannot be meaningfully summarized by a single average effect derived from publication counts. Observed estimates largely reflect variation across disciplines, institutional settings, career stages, and caregiving regimes. Accordingly, the meta-analysis should be interpreted as a diagnostic assessment of prevailing productivity metrics rather than as a definitive synthesis of parenthood-related penalties or premia.

To move beyond these limitations, the next section introduces original survey evidence capturing multiple dimensions of productivity. This allows us to contrast observable output with performance under constraint and perceived work effectiveness among academic parents and non-parents.

### Survey evidence: Objective and subjective productivity

#### Objective productivity.

**Fig 1:**
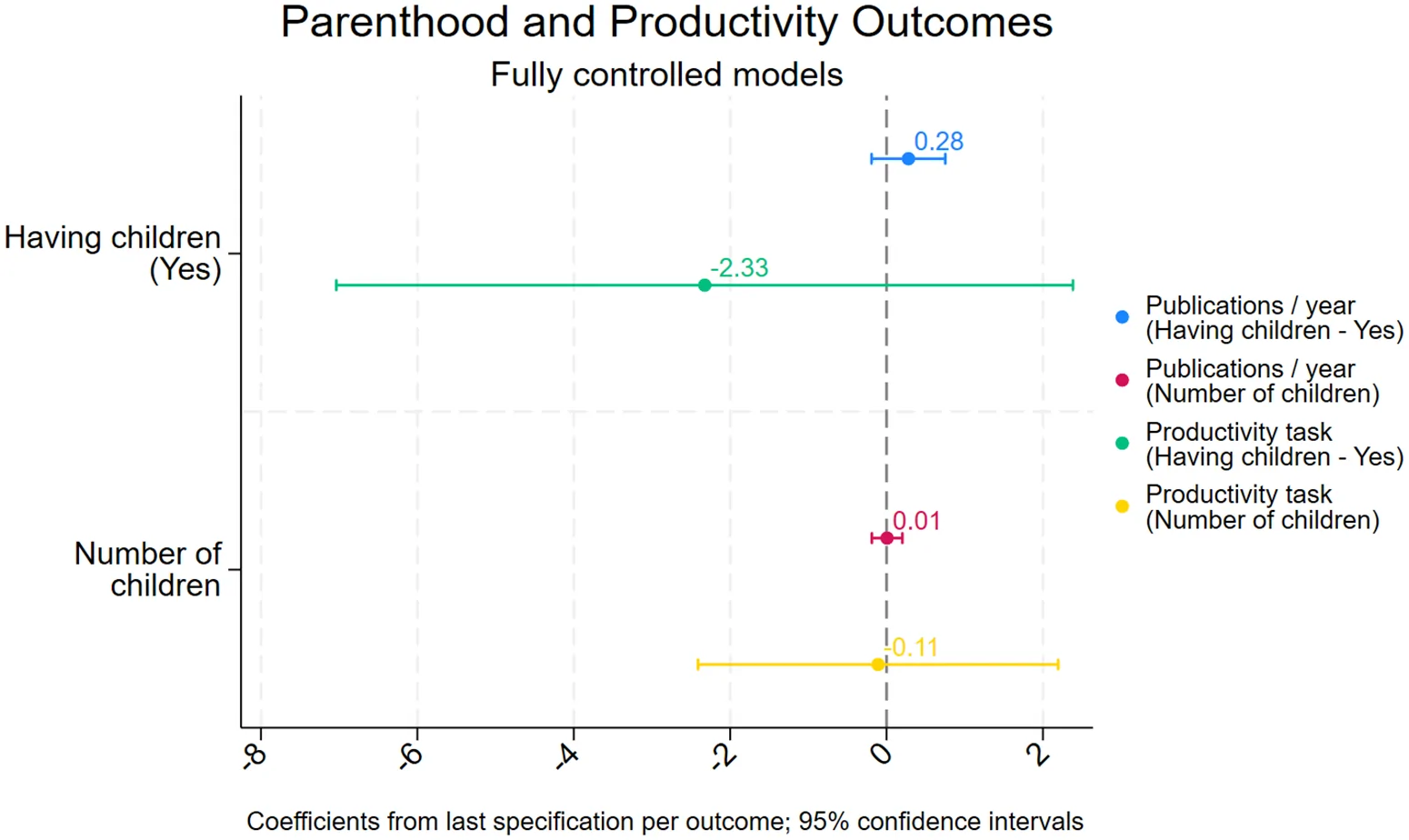
Parenthood and productivity outcomes (in fully controlled models). The figure reports coefficient estimates for parenthood measured as a binary indicator (having children) and as the number of children, across two objective productivity outcomes: publications per year and performance in a real-effort task. Points denote estimated coefficients and horizontal bars indicate 95% confidence intervals. All specifications include controls for gender, age and age squared, fertility preferences, working conditions, income and caregiving arrangements.

S1, S2, S3 and S4 Tables in the Supporting information report the results from the stepwise regression models examining the association between parenthood and academic productivity across alternative outcomes and operationalizations of parenthood. Fig 1 summarizes the estimated coefficients from the fully controlled specifications for each outcome.

Across all specifications, we find no robust association between parenthood and publications per year. When parenthood is measured using a binary indicator, the estimated coefficients are small and statistically significant in the baseline specifications; however, their magnitude decreases and they become statistically indistinguishable from zero as progressively richer sets of controls are introduced. These include demographic characteristics, working conditions, income, and caregiving-related variables, as well as fertility preferences, which serve to partially account for self-selection into parenthood and for unobserved differences in career orientation and life-course planning [17]. Following the suggestion of an anonymous reviewer, we also re-estimated the models controlling for respondents’ country of residence; the results remained substantively unchanged.

An identical pattern emerges when parenthood is operationalized as the number of children, with point estimates consistently centered around zero across specifications. Results for the real-effort task mirror those obtained for publication-based productivity: neither the binary indicator for parenthood nor the number of children is robustly associated with task performance once controls are taken into account.

This absence of statistically significant associations is robust across a wide range of alternative specifications and sensitivity analyses reported in S5 Table in the Supporting information. In particular, results remain unchanged when censoring outliers, when redefining productivity using a binary indicator for publishing at least one paper per year estimated via Logit or Probit models, and when also controlling for individual effort as captured by performance in the real-effort task.

To assess gender heterogeneity, we estimate interaction models including the full set of controls (S6 and S7 Tables). Across both productivity outcomes, interaction terms between parenthood and gender are small and statistically indistinguishable from zero, indicating no systematic gender differences in the association between parenthood and objective productivity.

S8 and S9 Tables in the Supporting information report results from panel data fixed-effects models estimated on a subsample of the full survey population consisting of respondents (N = 56) who voluntarily provided identifiable scholarly information, namely their ORCID iD and/or Google Scholar profile. Given the relatively small sample size, the statistical significance of these estimates should be interpreted with caution. Nevertheless, we report these analyses because they provide complementary evidence and additional insights that contribute to the interpretation of the main findings. The information provided by these respondents allowed us to reconstruct individual publication histories and build a longitudinal dataset aligned with the timing of the transition to parenthood. For each individual in this subsample, we collected the complete record of publications and their dates of appearance by scraping ORCID iD and/or Google Scholar profile. Using the reported year of birth of the children, we then reconstructed a person-year panel centered on the transition to parenthood, enabling us to track within-individual changes in productivity before and after childbirth. In addition to publication counts, we also derived a measure of average publication quality by linking each paper to external journal-level quality indicators drawn from the dataset described in the SCImago Journal and Country Rank dataset (https://github.com/ikashnitsky/sjrdata), which provides standardized measures of journal impact and quality over time (alternative empirical approaches [18,19]).

Results in S8 Table indicate a positive contemporaneous association between having had a child and publication output in the pooled sample and among men, while estimates for women are negative and statistically insignificant. However, once lagged indicators of parenthood are introduced (S9 Table), coefficients become statistically indistinguishable from zero across all specifications. This attenuation suggests that any short-run changes in productivity around childbirth do not persist over time and do not translate into systematic within-individual gains.

S10 Table reports panel estimates examining the association between parenthood and alternative indicators of publication quality, including the h-index, journal quartile, and journal rank. Across all three outcomes, coefficients on parenthood are small, imprecisely estimated, and statistically indistinguishable from zero in the pooled sample as well as when models are stratified by gender. These findings should be interpreted with caution given the heterogeneity in career positions, academic disciplines, and institutional contexts.

#### Subjective productivity losses.

Table 1 reports regression models examining correlates of self-reported productivity loss among parents. The outcome measures perceived reductions in work performance over the previous seven days on a 0–10 scale, with higher scores indicating greater impairment. Consistent with the original WPAI instrument, we adopted a 7-day reference period as a pragmatic compromise between minimizing recall bias associated with longer retrospective windows and avoiding the capture of highly specific or episodic experiences that may characterize shorter recall periods. As this outcome is measured only among respondents with children, the analysis is restricted to parents; accordingly, the estimated associations capture heterogeneity within the parent population rather than differences between parents and non-parents.

**Table 1:** Self-reported productivity loss from parenthood.

	(1)	(2)	(3)	(4)	(5)	(6)	(7)
Woman	0.919^*^	0.567	0.490	0.398	0.394	0.374	−0.462
Age		0.271^*^	0.306^**^	0.299^*^	0.278^*^	0.309^**^	0.391^***^
Age sq.		−0.004^**^	−0.004^***^	−0.004^***^	−0.004^**^	−0.004^**^	−0.005^***^
Ideal number of children			0.396	0.493	0.459	0.524	0.309
Remote work (yes)				0.831	0.728	0.858	0.426
Part-time					−0.712	−0.835	−0.715
Income (scale)						−0.144	−0.139
Weekly hours of childcare							0.021
Diff. in childcare hours							0.025^***^
Diff. in childcare hours sq.							0.000^**^
Constant	3.421^***^	−0.659	−2.024	−2.618	−1.767	−1.896	−4.036
Observations	104	104	101	99	99	95	93
*R* ^2^	0.033	0.151	0.178	0.193	0.205	0.213	0.351

Standard errors in parentheses. ^*^ *p* < 0.10, ^**^ *p* < 0.05, ^***^ *p* < 0.01.

Across specifications, women initially report higher perceived productivity loss; however, this association attenuates and becomes statistically insignificant once age, fertility preferences, work arrangements, and caregiving intensity are included. Age is positively associated with perceived productivity loss, with a concave pattern indicated by the negative coefficient on age squared, suggesting that perceived impairment increases with age at a decreasing rate.

**Fig 2:**
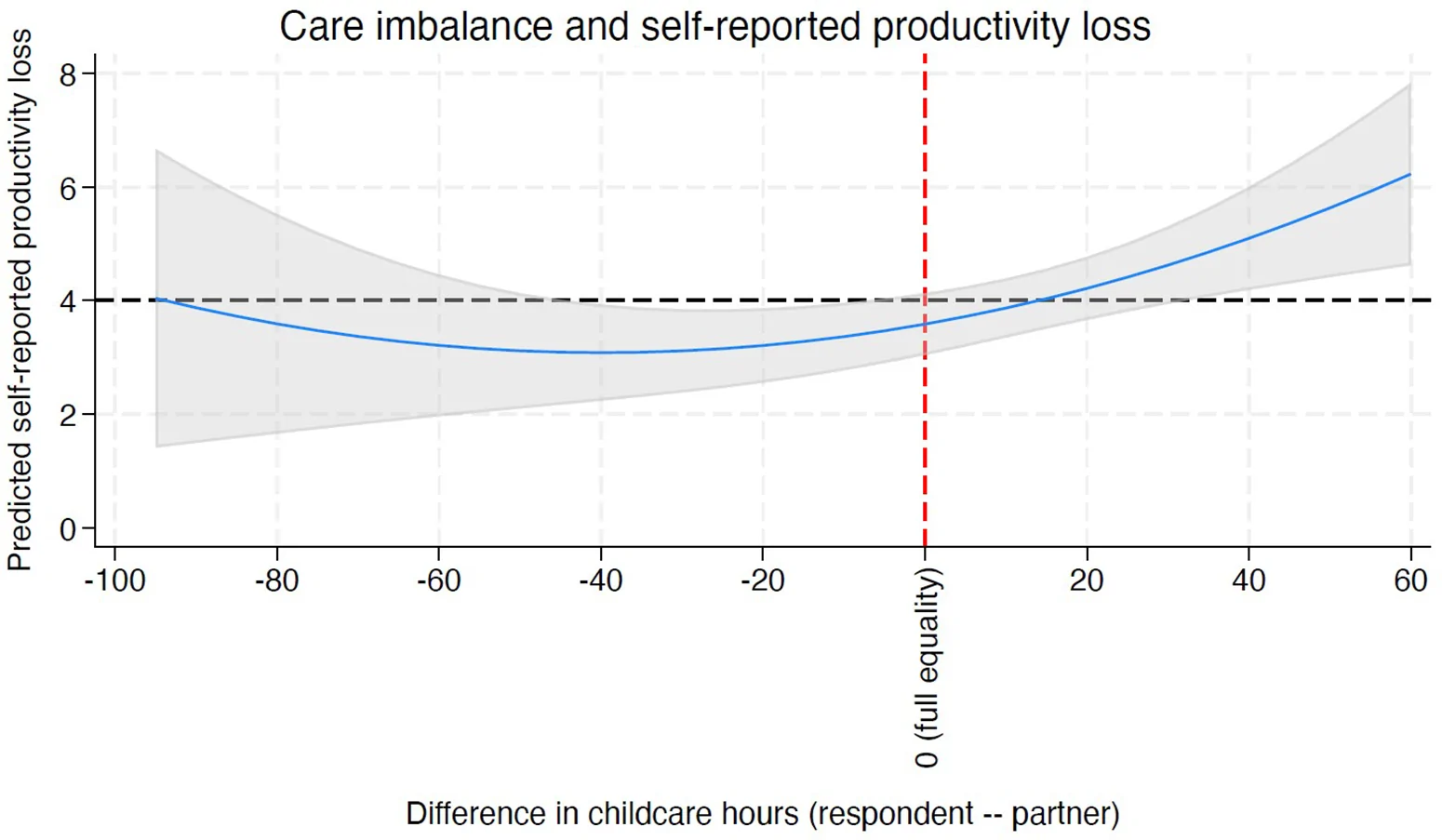
Care imbalance and self-reported productivity loss. The figure reports predicted values of self-reported productivity loss as a function of the within-couple difference in weekly childcare hours (respondent minus partner), based on a quadratic specification. The dashed vertical line at zero denotes full equality in childcare responsibilities, while the horizontal dashed line marks the mid-point of the productivity loss scale. Shaded areas represent 95% confidence intervals.

Fig 2 illustrates the relationship between within-couple childcare imbalances and self-reported productivity loss based on the quadratic specification. Given the importance of caregiving imbalance for our analysis, the questionnaire included separate items asking respondents to report the number of childcare hours performed by themselves and by their partners. Predicted productivity losses are lowest around a near-equal allocation of childcare and increase as the imbalance widens in either direction, with a markedly steeper gradient when respondents bear a substantially larger share of caregiving.

The estimated turning point of the quadratic function lies at a negative value of the imbalance index (approximately −40 hours) and is marginally significant (*p* = 0.057). This pattern indicates that productivity costs rise disproportionately when respondents assume a dominant caregiving role, whereas small deviations from equality are not associated with higher perceived losses.

Taken together, Fig 2 and the regression results indicate that perceived productivity impairment is driven less by caregiving per se than by asymmetric caregiving arrangements.

To test whether the association between childcare imbalances and perceived productivity loss varies by gender, we estimate models interacting gender with both the linear and quadratic terms of the care imbalance index. Joint tests reject the null of equal coefficients across genders (*F*(2,80)=4.97, *p* = 0.009), indicating significant gender heterogeneity in this relationship (S11 Table in Supporting information).

**Fig 3:**
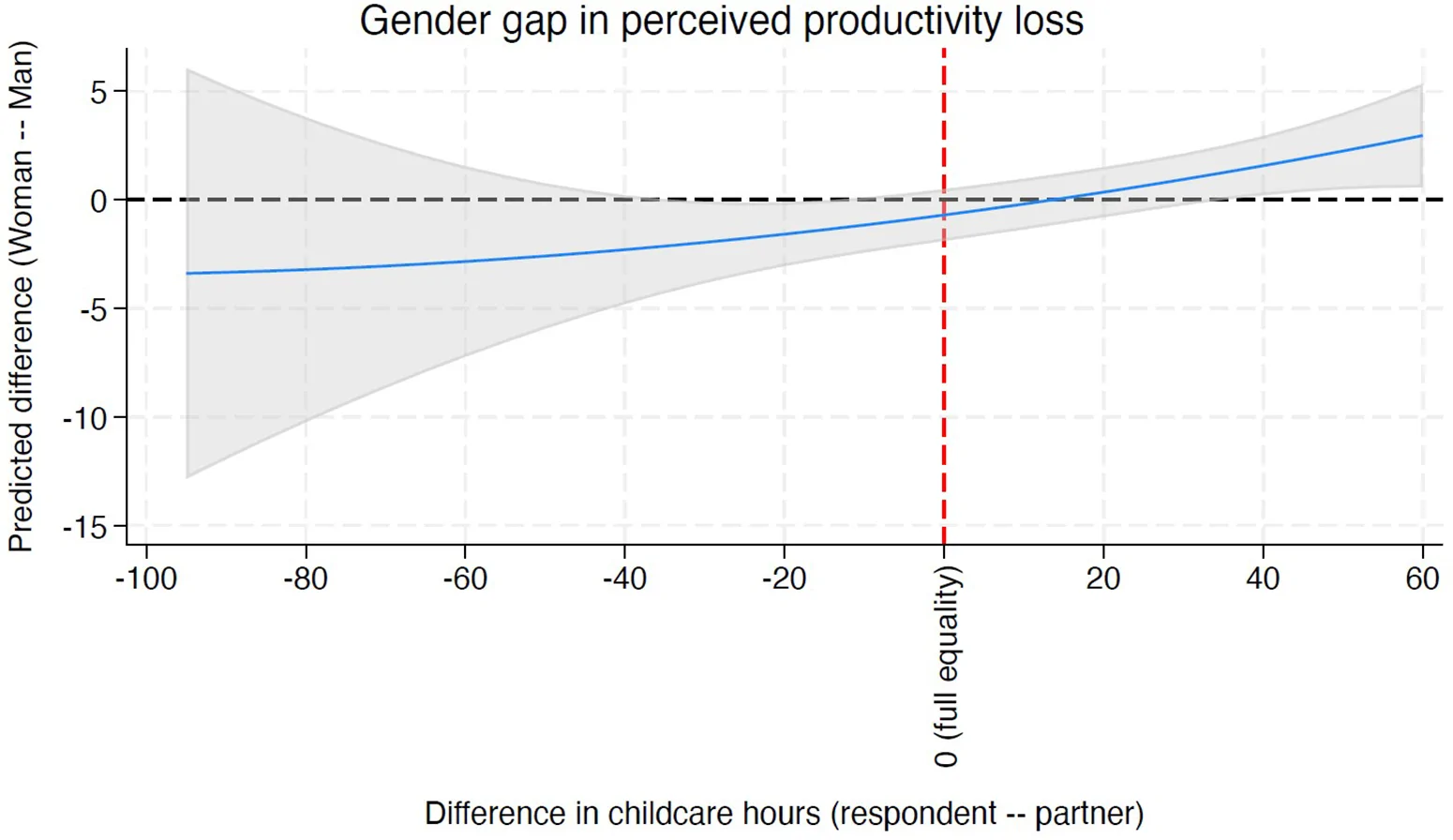
Gender gap in perceived productivity loss. The figure plots the predicted difference in self-reported productivity loss between women and men (Woman – Man) across values of the childcare imbalance index. Positive values indicate higher perceived productivity loss among women relative to men. The vertical dashed line marks full equality in childcare hours, and shaded areas represent 95% confidence intervals.

Fig 3 illustrates this heterogeneity by plotting the predicted difference in perceived productivity loss between women and men across the range of childcare imbalances. The gender gap is close to zero under near-equal allocation but becomes increasingly negative as the imbalance shifts toward women performing more childcare, indicating higher perceived losses among women. When men perform a larger share of childcare, the gap narrows and becomes statistically indistinguishable from zero.

Together, the regression and graphical evidence indicate that gender differences in perceived productivity loss arise primarily under asymmetric caregiving arrangements rather than reflecting a uniform gender penalty associated with parenthood. At the same time, the coexistence of relatively limited differences in objective productivity measures and substantially higher perceived productivity loss among women may suggest the presence of compensatory mechanisms. One possible interpretation is that women maintain comparable levels of observable output, thereby preserving measured productivity despite experiencing a stronger subjective sense of productivity loss. This interpretation is consistent with the idea that unequal caregiving burdens may affect perceived well-being and work pressure even when standard productivity indicators do not capture corresponding declines in output.

We next examine how respondents perceive the implications of parenthood along three dimensions: whether the experience of parenthood represents an advantage in their work, whether becoming a parent has changed their self-perception as a worker, and whether it has altered how colleagues perceive them as workers. Each outcome is measured using a three-category response scale (Yes / No / I don’t know) and analyzed using multinomial logit models with “I don’t know” as the reference category. Coefficients therefore capture the likelihood of expressing a definite evaluation—positive or negative—relative to uncertainty.

**Fig 4:**
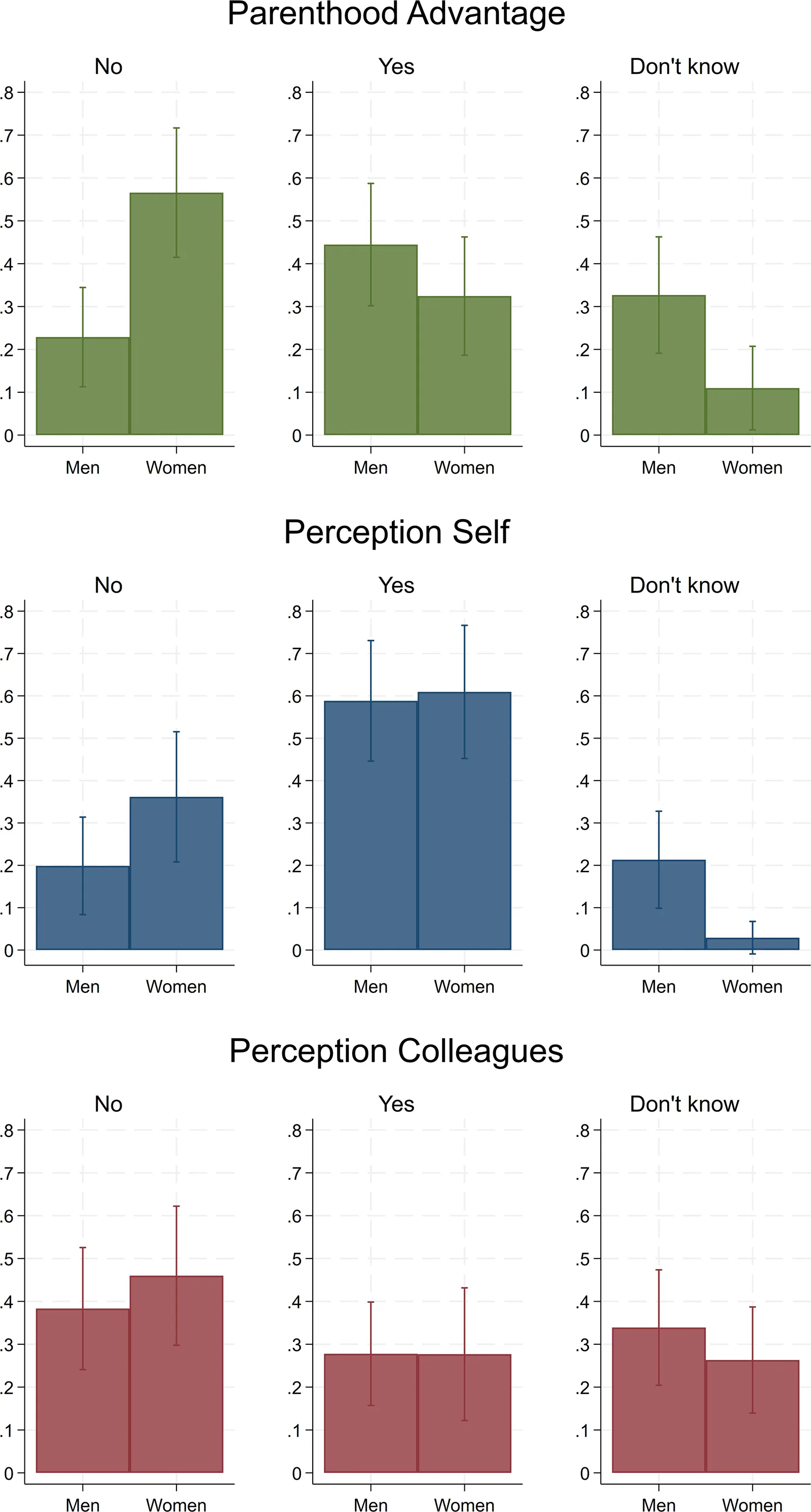
Predicted probabilities by gender for perceptions of parenthood. The figure reports predicted probabilities from multinomial logit models for three outcomes: whether parenthood represents a work-related advantage (top row), whether it has modified respondents’ self-perception as workers (middle row), and whether it has modified colleagues’ perceptions (bottom row). Predictions are computed holding covariates at their observed values. Error bars represent 95% confidence intervals.

Fig 4 displays predicted probabilities from the multinomial logit models reported in S12 Table in the Supporting information, disaggregated by gender and response category. The figure reveals clear gender differences in subjective evaluations of parenthood, which are most pronounced for internal assessments rather than perceptions of external recognition.

When asked whether parenthood represents a work-related advantage, women show a substantially higher probability of responding negatively and a lower likelihood of expressing uncertainty, whereas men are more likely to report either a positive assessment or uncertainty. A similar pattern emerges for self-perception as a worker: although affirmative responses are common among both genders, men are considerably more likely to select the “I don’t know” category, while women tend to provide more definite evaluations. In contrast, gender differences largely disappear when respondents assess how they are perceived by their colleagues. Predicted probabilities for men and women overlap across response categories, indicating that the consequences of parenthood are more salient in internalized interpretations of work identity than in perceived external evaluations.

To complement the quantitative analysis, we analyze open-ended responses on parenthood and work. After cleaning and tokenizing the texts, we compare word frequencies across respondents who view parenthood as a work-related advantage versus those who do not, and across self- and peer-related perceptions.

A clear lexical contrast emerges. Academics who reject parenthood as an advantage emphasize *work* and *time*, frequently in conjunction with *child*, *care*, and *responsibilities*, highlighting perceived tensions between caregiving demands and the time-intensive nature of academic research and teaching. By contrast, those who affirm an advantage use similar core terms but frame them differently, associating *work* and *time* with *focus*, *productivity*, *development*, and *perspective*, suggesting a reinterpretation of constraints as sources of efficiency and personal growth.

These frames are mirrored in accounts of self-perception. Respondents reporting changes after parenthood stress a reordering of priorities, with frequent references to *family*, *life*, and *now*, alongside continued attention to *work* and *time*. Parenthood thus appears to reshape how academic work is valued and organized rather than uniformly altering perceived capacity. Finally, perceived changes in colleagues’ views are ambivalent: terms indicating reduced *availability* and *commitment* coexist with positive attributions such as *responsibility*, *credibility*, and *trust*, pointing to shifting social evaluations of academic parents beyond productivity alone.

## Discussion

This paper asked a simple but consequential question: what do we actually learn about the relationship between parenthood and academic productivity when we look across measures, designs, and evaluative vantage points? Our answer is threefold. First, a set of small-scale meta-analyses of publication-based outcomes fails to yield a stable, generalizable effect of parenthood across studies, contexts, and groups; pooled estimates are statistically indistinguishable from zero and characterized by extreme heterogeneity. While publication-based indicators may overlook some dimensions of academic work, the absence of consistent effects could also reflect the genuinely context-dependent nature of parenthood effects, or the possibility that parenthood is not a primary determinant of productivity differences in the studies included in the meta-analysis. At the same time, null effects may also arise because publication counts are less sensitive to factors such as time constraints, cognitive load, and invisible academic labor, which may shape academic experiences without necessarily translating into measurable differences in output.

While stylized, this task captures core components relevant to academic work, including sustained attention, time management, and performance under pressure. It should therefore be interpreted as a measure of short-term effort-based performance under controlled conditions, rather than as a direct proxy for scholarly output. Tasks of this type are widely used in the experimental and behavioral economics literature as standardized measures of effort provision and productivity under incentives and time constraints. Within our framework, this measure complements publication-based indicators. While publications capture realized academic output over longer horizons and are shaped by institutional and collaborative factors, the real-effort task provides a controlled and comparable measure of individual performance under constraint. Taken together, the two approaches allow us to capture distinct but complementary dimensions of productivity. Second, original survey evidence and a complementary real-effort task show no robust association between parenthood and objective productivity, even under rich statistical control and in panel fixed-effects models that leverage within-individual variation. The results concerning academic productivity in our survey analysis differ from some of the context-specific findings reported in the studies included in the meta-analysis. One possible explanation for this discrepancy lies in the broader and more heterogeneous nature of our sample. Whereas many of the studies included in the meta-analysis focus on specific disciplines, institutions, or national academic systems, our survey encompasses 298 academics working across North American, European, and extra-European contexts.

Our findings therefore suggest that the productivity consequences of parenthood may be highly contingent upon contextual factors, and that generalizing results from single-country or single-discipline studies should be approached with caution.

Third, subjective and interpretive dimensions—self-reported productivity loss and perceptions of parenthood as a work-related (dis)advantage—are patterned by caregiving arrangements and, in key cases, by gender.

Taken together, these findings suggest that debates over “penalties” or “premia” associated with parenthood in academia cannot be adjudicated by publication counts alone. Instead, *evaluation regimes*—the social and institutional practices through which productivity is inferred, signaled, and rewarded—mediate how caregiving constraints translate (or fail to translate) into observable output. Our results contribute to a growing body of work that reframes productivity as a social and organizational construct rather than a neutral summary of accomplished tasks [12,20,21]. In this reframing, parenthood operates as a stress test: it exposes a structural disconnect between what is *measured* and what is *lived*.

Across specifications, parenthood is not robustly associated with annualized publications or performance in a time-pressured accuracy task. The null results hold when operationalizing parenthood as a binary indicator or as the number of children, after conditioning on demographic, occupational, and caregiving covariates, and in panel fixed-effects models built from reconstructed publication histories. This triangulation aligns with mixed and context-dependent evidence in the literature, including short-run spikes or slowdowns that attenuate over time and vary by institutional support and division of care [7,9,22]. In our view, the coherence of these nulls across designs is itself a substantive result: it cautions against treating publication counts as the privileged arbiter of “true” productivity in the presence of heterogeneous constraints and compensations.

In contrast to objective outcomes, self-reported productivity loss among parents rises with within-couple childcare imbalance and exhibits a non-linear profile, with the lowest perceived loss around near-equal allocation and steeper increases when respondents shoulder a dominant share of care. Interaction models indicate gender heterogeneity: perceived loss is disproportionately higher for women when they perform more childcare relative to their partners. This pattern is consistent with research documenting the asymmetric organization of care and its implications for career continuity and perceived performance [10,13,23]. The greater perceived productivity loss observed among mothers, despite relatively limited differences in objective productivity measures, may reflect compensatory mechanisms through which women maintain observable output at the cost of increased effort, time compression, or higher psychological strain.

Crucially, these are not merely “attitudes”: they are *evaluative experiences*—how individuals register constraints and reallocate effort as they negotiate academic workloads under care.

Multinomial models and the analysis of open-ended text show that gender differences are especially pronounced for *internalized* interpretations of work identity (e.g., whether parenthood is a work advantage; self-perception as a worker), while differences largely attenuate when respondents assess *colleagues’* perceptions. Women are significantly more likely than men to provide a definitive evaluation—most often selecting *No* rather than *I don’t know*—when assessing whether parenthood constitutes an advantage at work, potentially reflecting greater direct exposure to the “maternal wall” mechanisms that may hinder women’s career progression after becoming mothers [24,25]. This pattern contrasts with a large behavioral and experimental literature in which women are more likely to express uncertainty, opt out of competition, or avoid decisive choices under ambiguity, while men tend to exhibit greater decisiveness or overconfidence [26–29].

The divergence suggests that gender differences in uncertainty are context-dependent. In settings involving lived constraints, care responsibilities, and identity-based evaluation—rather than probabilistic beliefs or competitive performance—women may possess greater experiential clarity, while men are more likely to retain uncertainty. In this sense, decisiveness in our setting appears to reflect differential exposure to constraints rather than differences in confidence or ability.

Textually, academics who deny that parenthood is an advantage use a lexicon centered on *work*, *time*, *care*, and *responsibilities*, emphasizing the incompatibility between intensive caregiving and research/teaching demands. Those who affirm an advantage pair similar core terms with *focus*, *productivity*, *development*, and *perspective*, indicating a reinterpretation of constraints as catalysts for efficiency or growth. This divergence underscores that parenthood does not merely reallocate hours; it reshapes how productivity is *framed*, by whom, and with what consequences for recognition and self-assessment [6,12].

Our central implication is that the apparent inconsistency of parenthood effects reflects a mismatch between *metrics* and *mechanisms*. Output indicators are weakly responsive to heterogeneous care constraints (and compensations) that are partially absorbed through time-shifting, task reprioritization, collaboration strategies, and identity work. Meanwhile, evaluative practices—from self-appraisal to colleagues’ inferences about commitment and availability—are sensitive to care intensity and its distribution, with gendered patterns most visible where care is unequal. This helps reconcile three stylized facts: (i) nulls on publications; (ii) strong subjective productivity costs under care imbalance; (iii) polarized narratives about (dis)advantage.

If institutions want evaluation to reflect *productivity under constraint*, not just *output under ideal conditions*, three design principles follow. First, *diversify* the portfolio of recognized contributions beyond publication counts (e.g., mentoring, collaborative infrastructure, community and institutional service), particularly in stages where caregiving intensity peaks [11]. Second, *de-bias availability heuristics*: reduce the weight placed on physical presence and continuous schedulability when inferring commitment. Third, *track and mitigate care imbalance risks* by offering flexible workload allocation, predictable scheduling, high-quality childcare, and leave policies that do not penalize cumulative profiles [8,30]. None of these proposals require lowering standards; they require aligning what is counted with conditions under which work is done.

Our analyses are not designed for causal identification of parenthood effects, nor to disentangle potential heterogeneity across academic disciplines, research fields, or publication cultures. The meta-analyses are constrained by reporting standards and indicator heterogeneity in the underlying literature; our survey, while multi-sourced, is not nationally representative; the panel fixed effect (FE henceforth) exercise relies on a subsample with identifiable scholarly profiles. We also cannot fully observe within-institution workload, informal support, or hidden collaboration that buffers constraints. In addition, the relatively limited number of studies included in the meta-analysis and the heterogeneity of the available information do not allow for robust subgroup analyses by factors such as research intensity, academic discipline, career stage, country, or institutional prestige. As a result, potentially context-specific parenthood penalties in highly competitive academic environments may remain partially obscured. Conversely, stronger inferences could be drawn from a larger and more contextually homogeneous pool of studies. Nevertheless, the number of studies satisfying our inclusion criteria and reporting sufficiently comparable quantitative evidence is currently limited, constraining the scope for more fine-grained subgroup analyses.

These limitations are mitigated by triangulation across methods and outcomes, but they underscore that our claims are about coherence across measures, not causal magnitudes. However, the results should be interpreted primarily in light of the specific data, measures, and research design employed in the analysis, particularly given the partially unobservable heterogeneity across research disciplines and institutional contexts.

Accordingly, the findings may suggest several directions for future research, although these considerations should be interpreted cautiously given the heterogeneity of institutional and disciplinary contexts represented in the sample. First, future studies could further explore alternative approaches to productivity measurement by considering dimensions such as effort under constraint, temporal volatility, and team-based complementarities (e.g., coordination, mentoring, or data stewardship). Second, additional research may help clarify how local institutional arrangements — including parental leave policies, childcare availability, and workload allocation practices — relate to divergences between objective and subjective productivity indicators, potentially leveraging quasi-experimental variation where feasible. Third, a life-course perspective could provide further insight into how transitions into and out of intensive caregiving phases are associated with changes in output composition, collaboration patterns, and evaluative processes over time.

## Conclusions

In conclusion, this paper advances the debate on parenthood and productivity by shifting focus from the search for a single pooled effect to the alignment (or misalignment) between what productivity measures capture and how productivity is evaluated and lived in academic settings. We show that output indicators (publications, task performance) do not exhibit robust associations with parenthood once observable characteristics and caregiving covariates are accounted for, including in within-person designs. At the same time, perceived productivity costs rise with care imbalance in a non-linear and gendered fashion, and qualitative accounts reveal opposing frames—constraint versus growth—through which academics interpret the work–care interface.

The implication is not that parenthood is inconsequential for productivity, nor that evaluation is merely biased opinion. It is that prevailing output metrics are only weakly sensitive to the mechanisms through which care shapes work. At the same time, given the substantial heterogeneity across institutional settings, disciplinary cultures, and career stages observed in the literature, these implications should be interpreted with caution and not as universally applicable across all academic contexts. Institutions that take equity and excellence seriously should therefore reconsider how evaluation aggregates contributions and infers productivity from availability and presence. However, the specific form such policies may take will likely depend on institutional arrangements, national welfare systems, disciplinary expectations, and the organization of care within different academic environments. Policy levers that target distribution of care, recognition of undercounted work, and de-biasing of commitment signals are likely to have higher returns than attempts to detect universal penalties or premia in publication counts. Reframing productivity as performance under societal constraints, rather than output under idealized conditions, offers a more accurate and more equitable basis for academic evaluation.

Ultimately, the contribution of our study is to show that apparent contradictions in the literature are not empirical noise but conceptual evidence: when metrics are misaligned with mechanisms, estimates will vary wildly. At the same time, part of this variability may also reflect genuine contextual differences in how parenthood interacts with academic work across institutional and cultural settings. Recognizing this opens a path beyond the penalty-versus-premium stalemate toward evaluation systems that better reflect how academic work is produced, sustained, and reproduced in the presence of care.

## Methods

### Meta-analysis

#### Search strategy and inclusion criteria.

To provide a systematic assessment of the existing empirical evidence, we conducted a structured literature search across three major scholarly databases: Scopus, Web of Science, and Google Scholar. Following established guidelines for systematic reviews [31], we used Boolean combinations of keywords related to parental status (e.g., “*parenthood*,” “*motherhood*,” “*fatherhood*,” “*children*,” “*gender*”) and academic productivity (e.g., “*university*,” “*published articles*,” “*peer-reviewed*”).

The exact search syntax deployed both in Scopus and Web of Science was structured as follows:

(TITLE-ABS-KEY (parenthood) AND TITLE-ABS-KEY (academia) AND TITLE-ABS-KEY (productivity) OR TITLE-ABS-KEY (motherhood) OR TITLE-ABS-KEY (fatherhood) OR TITLE-ABS-KEY (university) OR TITLE-ABS-KEY (child)) AND PUBYEAR > 2010 AND PUBYEAR < 2025

The search initially identified 76 potentially relevant studies published between 2011 and 2025. To ensure methodological and contextual comparability across the included evidence, we applied a set of predefined inclusion criteria. First, we restricted the sample to studies conducted in Western countries, as these share broadly comparable academic systems, institutional contexts, and career structures. In addition, we excluded studies reporting only qualitative findings, as they did not provide quantitative estimates suitable for inclusion in the analysis. To ensure methodological consistency across studies, we included only those that measured academic productivity through observable research outputs, namely peer-reviewed journal articles, books, book chapters, and publications in professional journals. Studies relying on alternative indicators such as citation counts, income, working hours, grant success, or subjective prestige measures were excluded. In particular, citation counts are highly field- and time-dependent, reflecting visibility and cumulative advantage processes rather than contemporaneous productivity alone. Grant success and income are strongly influenced by funding systems, institutional prestige, and national academic structures, while working hours measure effort allocation rather than observable scholarly production. Subjective prestige indicators, moreover, introduce substantial cross-study heterogeneity due to differences in self-perception and evaluation criteria. By focusing exclusively on observable publication outputs, we aimed to reduce measurement heterogeneity and ensure that the meta-analysis compared studies relying on relatively consistent and directly observable productivity metrics. At the same time, this selective criterion may have influenced the results obtained, as studies capturing broader dimensions of academic performance—such as citations, grants, income, or prestige—were excluded. However, including these heterogeneous indicators would likely have reduced the reliability and comparability of the pooled estimates, given the substantial variation in how such outcomes are measured across disciplines, institutions, and national contexts. The application of the aforementioned exclusion criteria removed 39 studies that were not directly relevant to the scope of the analysis.

In a second step, we excluded 29 ineligible studies that focused exclusively on gender differences without accounting for parental status, as well as those examining only one gender among parents, as these designs did not allow for meaningful comparative analysis, incomplete statistical reporting, notably the absence of essential descriptive statistics (sample size, means, and standard deviations) or not available. Attempts to retrieve missing information by contacting the original authors were unsuccessful or yielded insufficient data for meta-analytic purposes.

**Fig 5:**
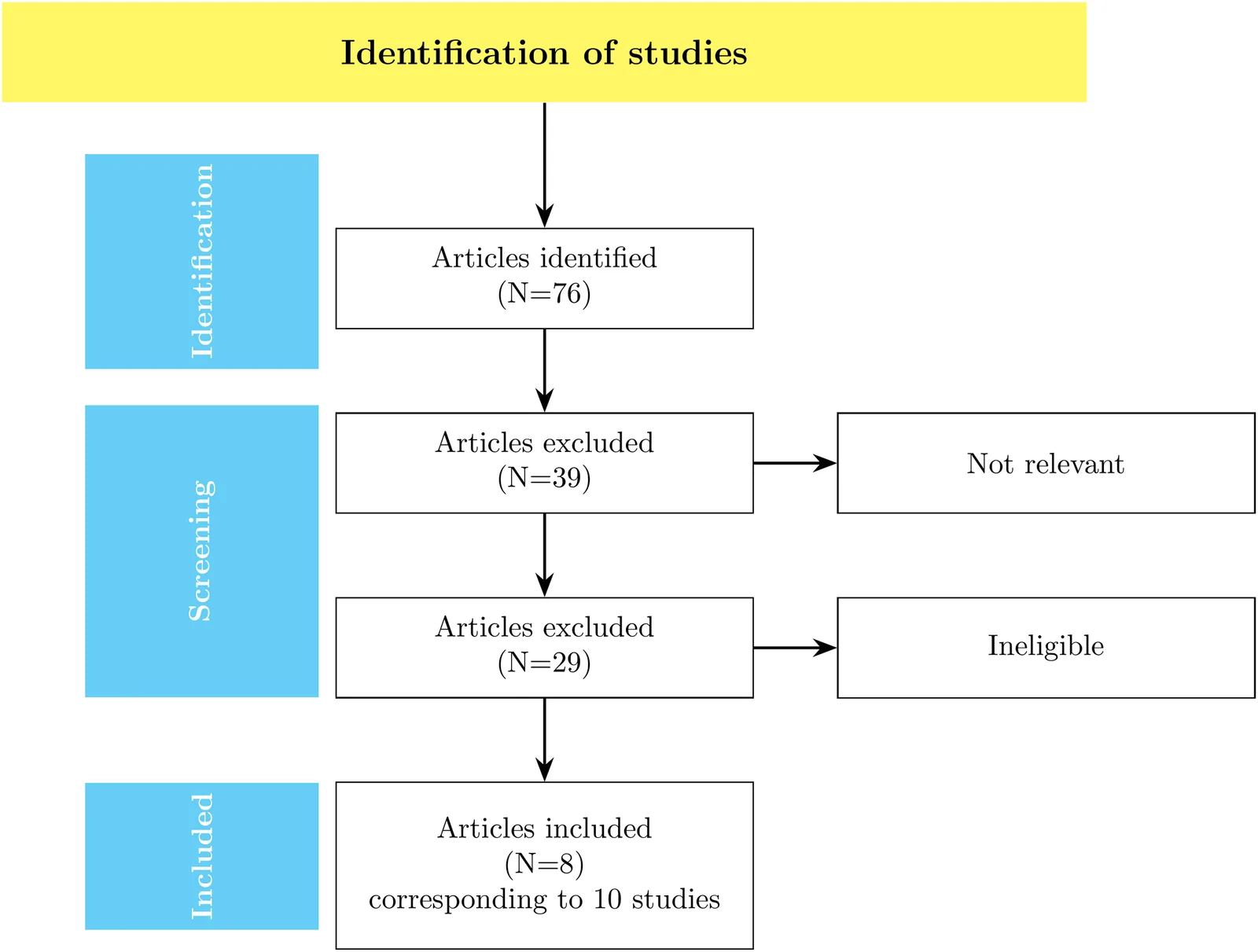
PRISMA flowchart.

The final dataset, therefore, consists of 8 studies meeting all inclusion criteria. One study was included multiple times because it reported results separately for distinct disciplinary subfields (computer science, business, and history), resulting in a total of 10 effect sizes. The full selection process is summarized in Fig 5. The excluded studies are summarized in S13 Table available in the Supporting information.

#### Studies included in the meta-analyses.

From the studies included in the meta-analysis (Table 2), we separately extracted productivity estimates for the six groups of interest: mothers, fathers, childless women, childless men, women, and men. This harmonized classification enables direct comparisons across parental and gender statuses while preserving the disciplinary, institutional, and geographical heterogeneity of the original studies.

**Table 2:** Studies included in the analysis.

ID	Title	Authors	Year	Country
1	Mothers of invention? Gender, motherhood, and new dimensions of productivity in the science profession [12]	Whittington, K. B.	2011	USA
2	Childbearing and (female) research productivity: A personnel economics perspective on the leaky pipeline [32]	Joecks, J., Pull, K., & Backes-Gellner, U.	2014	Germany, Austria, and German-speaking Switzerland
3	Enabling work? Family-friendly policies and academic productivity for men and women scientists [30]	Feeney, M. K., Bernal, M., & Bowman, L.	2014	USA
4	Is there a motherhood penalty in academia? The gendered effect of children on academic publications [33]	Lutter, M. & Schröder, M.	2020	Germany
5	The unequal impact of parenthood in academia [9]	Morgan, A. C., Way, S. F., Hoefer, M. J., Larremore, D. B., Galesic, M., & Clauset, A.	2021	USA and Canada
6	The unequal impact of parenthood in academia [9]	Morgan, A. C., Way, S. F., Hoefer, M. J., Larremore, D. B., Galesic, M., & Clauset, A.	2021	USA and Canada
7	The unequal impact of parenthood in academia [9]	Morgan, A. C., Way, S. F., Hoefer, M. J., Larremore, D. B., Galesic, M., & Clauset, A.	2021	USA and Canada
8	Gender, parenthood, and academic performance: Work-life and work-work balance in Russian academia [34]	Chechik, E.	2023	Russia
9	Parenthood premium but fatherhood super-premium in academic productivity? A matter of partner’s employment [8]	Tattarini, G., Gorodetskaya, O., & Vitali, A.	2024	Germany
10	Women in science: Lessons from the baby boom [14]	Kim, S. & Moser, P.	2025	USA

The evidence synthesized in the review is characterized by substantial heterogeneity, as many of the included studies rely on context-specific measures and focus on particular academic disciplines or institutional settings. Nevertheless, a predominant pattern emerges: most studies report a productivity penalty for mothers, both relative to childless women and to fathers. This finding suggests that the observed disadvantage is closely linked to the maternal role within the parenting process rather than to gender alone. Importantly, this motherhood penalty appears to be relatively consistent across diverse academic contexts. The studies documenting such disadvantages span a wide range of disciplines and countries, including sociologists in the German academic system, scholars in business, history, and computer science in North America, and economists in Russia.

A smaller subset of studies reports the opposite effect, identifying a parenthood premium, particularly for fathers. These findings are most frequently observed in German-speaking academic contexts.

Finally, research on scientists in the United States evidences mixed-effect, for example, indicates that mothers tend to be less productive than fathers during the years immediately following childbirth, but subsequently experience a recovery and, in some cases, surpass fathers in productivity during later career stages.

#### Estimation strategy.

From each study, we extracted information on sample sizes and publication outcomes expressed as mean values and associated standard deviations. When these statistics were not directly reported, effect sizes were derived from published regression estimates using standard conversion procedures. Table 3 summarizes the extracted data. Effect sizes were computed using Hedges’ *g*, which provides bias-corrected estimates and is particularly appropriate for small or unbalanced samples [35]. To assess between-study heterogeneity, we conducted formal tests of homogeneity. Across all specifications, the *I²* statistic exceeded 90%, indicating that the vast majority of observed variation reflects genuine differences across studies rather than sampling error.

**Table 3:** Descriptive statistics by group.

ID	Mothers			Childless women			Fathers			Childless men			All		Women		Men	
	N	Mean	SD	N	Mean	SD	N	Mean	SD	N	Mean	SD	Effect size	SE	Effect size	SE	Effect size	SE
1	2132	8.61	0.33	2745	7.10	0.26	10736	11.44	0.20	8226	10.55	0.23	–	–	–	–	–	–
2	–	–	–	–	–	–	–	–	–	–	–	–	0.023	0.014	0.104	0.056	0.110	0.160
3	–	–	–	–	–	–	–	–	–	–	–	–	0.063	0.107	−0.219	0.146	0.093	0.126
4	119	5.88	9.88	217	3.25	7.83	213	7.76	16.47	256	3.87	9.67	–	–	–	–	–	–
5	142	3.81	5.65	63	2.98	3.51	582	4.80	6.15	172	3.69	4.28	–	–	–	–	–	–
6	110	0.88	1.20	40	0.57	0.99	256	0.89	1.46	69	0.55	1.11	–	–	–	–	–	–
7	74	0.39	0.70	34	0.40	0.70	111	0.66	1.23	42	0.51	0.92	–	–	–	–	–	–
8	478	72.58	60.5	303	55.3	47.6	229	90.8	91.0	165	67.6	72.5	–	–	–	–	–	–
9	23	2.04	1.97	114	1.53	1.90	32	3.14	4.04	131	1.74	2.34	–	–	–	–	–	–
10	736	0.61	0.0576	2567	0.171	0.614	34320	0.291	0.88	11620	0.271	0.917	–	–	–	–	–	–

This high degree of heterogeneity, despite the stringent sample selection criteria adopted in the present review, may be explained by substantial variation across studies in disciplinary contexts, country settings, academic career stages (ranging from PhD students to senior professors), and the operationalization of productivity outcomes, including normalized indicators, cumulative publication counts, and annualized productivity measures. These dimensions are likely to constitute important sources of the observed between-study heterogeneity.

The corresponding homogeneity tests were statistically significant (*p* < 0.001), confirming substantial heterogeneity. Given this degree of variability, we adopted a random-effects framework estimated via Restricted Maximum Likelihood (REML), allowing true effect sizes to vary as a function of contextual, methodological, and sample-specific factors. Finally, we assessed potential publication bias using Egger’s regression test [36], which examines whether estimated effect sizes are systematically related to their precision.

## Survey

### Data and empirical strategy

#### Sample and data collection.

The sample employed in this study was obtained using two complementary recruitment strategies. The first relied on the online research platform Prolific (https://www.prolific.com), which is widely recognized in the academic literature as a reliable and efficient tool for participant recruitment ([37–39]). The second consisted of a stratified sampling procedure conducted through online social networks (i.e., LinkedIn). All participants completed the same custom-designed questionnaire.

While the Prolific platform prohibits the collection of personally identifiable information, respondents recruited through online social networks were invited, on a fully voluntary basis, to provide identifiable scholarly information in the form of ORCID iDs and/or Google Scholar profiles. These identifiers enabled validation of self-reported publication data and allowed us to reconstruct detailed publication histories for a subsample of respondents. In accordance with ethical standards approved by the SUPSI Research Ethics Committee (Protocol No. CER-57513), the provision of such identifiers was entirely optional, given their potentially identifying nature.

Participants were recruited through the Prolific platform using a set of predefined eligibility and screening criteria. First, respondents were asked to indicate their current country of residence: individuals residing in the United States were included in the first wave of data collection, while those residing in other countries were assigned to the second sample. Second, only participants who self-reported a fluent level of English proficiency were eligible for inclusion; respondents indicating insufficient fluency were excluded. Third, participants were screened based on their occupational role: only those who were researchers, PhD students, postdoctoral fellows, university professors, or closely related academic positions were retained in the sample. Finally, respondents were asked about the type of institution in which they were employed; eligibility was restricted to individuals working in universities or university-affiliated medical or research centers.Using this recruitment strategy, we obtained a total sample of 218 participants, including 94 respondents residing in the United States and 124 residing in other countries (68 from Europe and 56 from outside Europe). This sample was further complemented by 80 additional respondents recruited through stratified sampling via social media, primarily based in Italy and Switzerland (Table 4). Among these respondents, 56 provided valid ORCID and/or Google Scholar identifiers, which constituted the analytical sample for the panel analysis.

**Table 4:** Sample composition.

	Prolific			Social Media		
	USA	Europe	Extra-Europe	USA	Europe	Extra-Europe
**Frequency**	94	68	56	1	78	1

The final analytic sample comprises 298 participants for which we have completed information on publication only for (252) and task (282), including 141 men, 153 women, and 4 individuals who did not disclose their gender identity. Among them, 110 respondents are parents (49 women and 61 men), while 188 are non-parents (104 women, 80 men, and 4 who prefer not to say). The average age of participants is 35.23 years, with parents being substantially older on average (42.78 years) than non-parents (30.82 years).

#### Measures of productivity.

We examine productivity using two objective measures. The first is *publications per year*, defined as cumulative scholarly output normalized by years of active research. The second is performance in a standardized *real-effort task*, widely used as standardized measures of performance and productivity under incentives and time constraints (i.e., [40–42]). In this task, participants were asked to count the number of zeros in a binary vector containing 103 zeros. Both the reported count and the time taken to complete the task (up to a maximum of 60 seconds) were recorded automatically.

Accuracy is defined as:


$$\begin{array}{c} \text{Accuracy}=\frac{\text{Zeros counted}}{103} \end{array}$$ (1)

Overall task-based productivity is computed as:


$$\begin{array}{c} \text{Productivity}=\text{Accuracy}\times \left(\frac{1000}{\text{Time taken}}\right) \end{array}$$ (2)

This measure rewards fast and accurate responses while penalizing both errors and longer completion times, providing a concise indicator of performance under cognitive and time constraints.

#### Parenthood and covariates.

Parenthood is operationalized in two ways: (i) a binary indicator distinguishing respondents with at least one child from those without children, and (ii) a continuous measure capturing the number of children, allowing for potential gradient effects in caregiving intensity. All models are estimated separately for these two operationalizations.

We adopt a stepwise modeling strategy. Baseline specifications include only the parenthood measure. We then progressively introduce controls for gender, age and age squared, fertility preferences (measured by the ideal number of children), working conditions (including access to remote work and employment status), and income. In the most comprehensive specifications, we additionally control for caregiving-related variables, including total hours of care provided by the respondent, the within-couple difference in caregiving hours, and its squared term, allowing for potential non-linear effects in the division of caregiving responsibilities.

To assess whether associations between parenthood and productivity differ by gender, we further estimate fully controlled models that include an interaction term between parenthood and gender. All models are estimated using ordinary least squares with heteroskedasticity-robust standard errors. Results are presented in a sequence of tables reporting the full set of specifications and are summarized graphically using coefficient plots with 95% confidence intervals.

#### Panel extension.

For the subsample of respondents who voluntarily provided ORCID and/or Google Scholar identifiers (N = 56), we reconstructed individual publication histories by collecting publication dates from these external sources. Using the reported year of childbirth, we constructed a person-year panel centered on the transition to parenthood and estimated fixed-effects models exploiting within-individual variation over time. In addition to publication counts, we linked each publication to external journal-level quality indicators to derive an average measure of publication quality. This panel analysis serves as a robustness and validation exercise rather than a causal design.

This empirical strategy is not intended to identify causal effects of parenthood on productivity. Instead, it is designed to examine the internal coherence—or divergence—of results across alternative productivity measures within a unified evaluative framework, while mitigating concerns related to self-selection into parenthood by conditioning on stated fertility preferences and, where possible, exploiting within-individual variation.

On average, participants report 13.83 published articles, with substantial differences by gender (18.78 among men versus 9.66 among women) and parental status (26.91 among parents versus 4.78 among non-parents), reflecting differences in age and career stage. Performance on the real-effort task averages 19.21, with modest differences by gender and parental status. As a reference point, completing the task correctly within 20 seconds would yield a score of 50.

## Supplementary Materials

### Meta-analysis

We conducted five small-scale meta-analyses to examine whether systematic differences in academic publication output can be identified by gender and parental status. Rather than testing for a single average effect, the analyses aim to assess the degree of convergence—or lack thereof—across the existing literature.

#### Women vs. men.

The pooled comparison between women and men in academia yields a negative but statistically non-significant effect size (Hedges’ *g* = –2.55, SE = 1.65, *p* = 0.12; 95% CI: [–5,79, 0.69]; S1 Fig). While the direction of the point estimate suggests lower publication output among women relative to men, the confidence interval includes the null value and the extremely high level of heterogeneity (*I²* = 99.98%) substantially limits the interpretability of the pooled effect.

#### Parents vs. non-parents.

The pooled comparison between parents and non-parents yields no statistically significant average effect (Hedges’ *g* = 0.85, SE = 0.54, *p* = 0.12; 95% CI: [−0.21, 1.91]; S2 Fig). While the point estimate is positive, the confidence interval is wide and includes zero, indicating that no robust conclusion can be drawn regarding either a productivity penalty or premium associated with parenthood. Egger’s test provides no evidence of publication bias. Heterogeneity, however, is extreme, suggesting that differences across studies overwhelmingly reflect contextual variation rather than random error (*I²* = 99.97%). Such dispersion implies that simple dichotomies between parents and non-parents mask critical dimensions, including career stage, disciplinary publication norms, and institutional support structures [43,44]. Overall, the absence of a statistically significant pooled effect indicates that parenthood does not exert a uniform influence on academic publication output. This finding reconciles prior evidence reporting both positive [8,9] and negative effects [6,11,33], and is consistent with longitudinal accounts documenting short-term increases followed by longer-term slowdowns in productivity [7].

#### Mothers vs. childless women.

The comparison between mothers and childless women similarly yields no statistically significant pooled effect (Hedges’ *g* = 0.64, SE = 0.51, *p* = 0.21; 95% CI: [—0.36, 1.63]; S3 Fig). Egger’s test does not indicate publication bias. Heterogeneity remains extremely high (*I²* = 99.72%), reinforcing the conclusion that motherhood effects are highly context-dependent. While some studies report positive or null associations [8,32], the broader literature documents negative impacts of motherhood on research output [5,10–12,22,33]. The lack of a robust pooled effect suggests that any advantages or penalties associated with motherhood are neither systematic nor consistently replicated across institutional settings.

#### Fathers vs. childless men.

The pooled comparison between fathers and childless men also fails to reach statistical significance (Hedges’ *g* = 0.61, SE = 0.40, *p* = 0.13; 95% CI: [–0.18, 1.39]; S4 Fig). Egger’s test again reveals no evidence of publication bias. As in previous analyses, heterogeneity is substantial (*I²* = 99.84%), indicating pronounced variability across studies. Although some contributions report a fatherhood-related productivity advantage [8,22], others find no meaningful differences [33]. The meta-analytic results suggest that any fatherhood premium is modest, context-dependent, and not robust across empirical settings.

#### Mothers vs. fathers.

The comparison between mothers and fathers yields a negative but statistically non-significant pooled effect (Hedges’ *g* = –1.72, SE = 1.54, *p* = 0.26; 95% CI: [–4.74, 1.29]; S5 Fig). While the point estimate is consistent with arguments emphasizing gendered divisions of care [23], the wide confidence interval and extreme heterogeneity (*I²* = 99.96%) preclude strong conclusions. Taken together with prior evidence [8,11,22], these findings suggest that observed gender differences in academic productivity among parents are highly sensitive to disciplinary norms, institutional arrangements, and the distribution of caregiving responsibilities.

## Supporting information

S1 Fig Hedges’ g Women vs. Men. (PNG)

S2 Fig Hedges’ g Parents vs. Non Parents. (PNG)

S3 Fig Hedges’ g Mothers vs. Childless women. (PNG)

S4 Fig Hedges’ g Fathers vs. Childless men. (PNG)

S5 Fig Hedges’ g Mothers vs. Fathers. (PNG)

S1 Table Publications per Year and Parenthood. (PDF)

S2 Table Publications per Year and Number of Children. (PDF)

S3 Table Effort Task and Parenthood. (PDF)

S4 Table Effort Task and Number of Children. (PDF)

S5 Table Robustness Checks. (PDF)

S6 Table Gender Interactions. (PDF)

S7 Table Interaction Between Gender and Difference in Hours of Care. (PDF)

S8 Table Parenthood and Productivity: Panel Data Fixed-Effects Model. (PDF)

S9 Table Parenthood and Productivity: Panel Data Fixed-Effects Model. (PDF)

S10 Table Parenthood and Publication Quality: Panel Data Fixed-Effects Models. (PDF)

S11 Table Self-reported Productivity Loss from Parenthood. (PDF)

S12 Table Perceptions of Parenthood (Multinomial Logit). (PDF)

S13 Table Excluded Studies. (PDF)
