# Supplementary material for: Parenthood and care in academia: Publication counts, self-assessed productivity, and gendered differences
Source: PLoS One. 2026 Sep 16;21(9):e0355683. doi: 10.1371/journal.pone.0355683 (PMC13580985; doi:10.1371/journal.pone.0355683)
Supplement: Supplementary file 1 [file pone.0355683.s006.pdf]

Table S1: Publications per Year and Parenthood

	(1)	(2)	(3)	(4)	(5)	(6)	(7)	(8)
Dummy Children								
No children	Ref.	Ref.	Ref.	Ref.	Ref.	Ref.	Ref.	Ref.
Yes children	0.572***	0.551***	0.329**	0.223	0.179	0.208	0.119	0.280
	(0.133)	(0.133)	(0.128)	(0.142)	(0.158)	(0.159)	(0.163)	(0.239)
Gender								
Man		Ref.	Ref.	Ref.	Ref.	Ref.	Ref.	Ref.
Woman		-0.142	-0.093	-0.117	-0.186	-0.187	-0.166	-0.103
		(0.132)	(0.140)	(0.149)	(0.170)	(0.170)	(0.166)	(0.170)
Age			0.018	0.031	0.034	0.020	-0.008	-0.022
			(0.061)	(0.062)	(0.066)	(0.061)	(0.071)	(0.075)
Age sq.			-0.000	-0.000	-0.000	-0.000	0.000	0.000
			(0.001)	(0.001)	(0.001)	(0.001)	(0.001)	(0.001)
Desired N. of Children								
Does not want children				Ref.	Ref.	Ref.	Ref.	Ref.
1 child				0.170	0.184	0.200	0.232	0.192
				(0.156)	(0.181)	(0.180)	(0.186)	(0.185)
2 children				0.316**	0.296*	0.314**	0.279*	0.248*
				(0.142)	(0.156)	(0.152)	(0.145)	(0.146)
3 or more children				0.175	0.181	0.184	0.145	0.133
				(0.145)	(0.161)	(0.160)	(0.165)	(0.169)
Dummy Remote Work								
No					Ref.	Ref.	Ref.	Ref.
Yes					0.376***	0.376***	0.363***	0.366***
					(0.130)	(0.126)	(0.122)	(0.125)
Dummy Type of Employment								
Full-time						Ref.	Ref.	Ref.
Part-time						-0.254	-0.151	-0.161
						(0.167)	(0.195)	(0.201)
Income (scale)							0.159*	0.160*
							(0.082)	(0.082)
Weekly hours of childcare								0.001
								(0.004)
Diff. in childcare hours								-0.006
								(0.004)
Diff. in childcare hours sq.								-0.000**
								(0.000)
Constant	0.490***	0.572***	0.010	-0.388	-0.668	-0.335	-0.513	-0.234
	(0.075)	(0.129)	(1.276)	(1.272)	(1.328)	(1.213)	(1.154)	(1.221)
Observations	252	249	249	240	218	218	214	212
R^2	0.074	0.078	0.099	0.108	0.119	0.128	0.154	0.166

Standard errors in parentheses * $p < 0.10$, ** $p < 0.05$, *** $p < 0.01$
