# Supplementary material for: Parenthood and care in academia: Publication counts, self-assessed productivity, and gendered differences
Source: PLoS One. 2026 Sep 16;21(9):e0355683. doi: 10.1371/journal.pone.0355683 (PMC13580985; doi:10.1371/journal.pone.0355683)
Supplement: Supplementary file 2 [file pone.0355683.s007.pdf]

Table S2: Publications per Year and Number of Children

	(1)	(2)	(3)	(4)	(5)	(6)	(7)	(8)
Number of children	0.270***	0.257***	0.107	0.054	0.029	0.041	0.008	0.007
	(0.067)	(0.070)	(0.067)	(0.077)	(0.082)	(0.081)	(0.086)	(0.099)
Gender								
Man		Ref.	Ref.	Ref.	Ref.	Ref.	Ref.	Ref.
Woman		-0.138	-0.095	-0.120	-0.187	-0.188	-0.168	-0.111
		(0.134)	(0.142)	(0.150)	(0.170)	(0.170)	(0.167)	(0.170)
Age			0.034	0.043	0.047	0.035	0.001	-0.013
			(0.061)	(0.063)	(0.067)	(0.063)	(0.072)	(0.077)
Age sq.			-0.000	-0.000	-0.000	-0.000	0.000	0.000
			(0.001)	(0.001)	(0.001)	(0.001)	(0.001)	(0.001)
Desired N. of Children								
Does not want children				Ref.	Ref.	Ref.	Ref.	Ref.
1 child				0.238	0.246	0.269	0.277	0.230
				(0.165)	(0.190)	(0.190)	(0.194)	(0.191)
2 children				0.375***	0.356**	0.378***	0.324**	0.315**
				(0.135)	(0.144)	(0.139)	(0.130)	(0.134)
3 or more children				0.233	0.247	0.252	0.200	0.206
				(0.155)	(0.173)	(0.172)	(0.180)	(0.185)
Dummy Remote Work								
No					Ref.	Ref.	Ref.	Ref.
Yes					0.390***	0.392***	0.373***	0.371***
					(0.131)	(0.127)	(0.122)	(0.125)
Dummy Type of Employment								
Full-time						Ref.	Ref.	Ref.
Part-time						-0.241	-0.142	-0.136
						(0.167)	(0.195)	(0.197)
Income (scale)							0.162**	0.164**
							(0.081)	(0.082)
Weekly hours of childcare								0.004
								(0.004)
Diff. in childcare hours								-0.007*
								(0.004)
Diff. in childcare hours sq.								-0.000*
								(0.000)
Constant	0.539***	0.620***	-0.273	-0.659	-0.953	-0.659	-0.743	-0.484
	(0.078)	(0.129)	(1.278)	(1.291)	(1.353)	(1.247)	(1.189)	(1.271)
Observations	252	249	249	240	218	218	214	212
R^2	0.061	0.066	0.089	0.103	0.116	0.124	0.152	0.162

Standard errors in parentheses * $p < 0.10$, ** $p < 0.05$, *** $p < 0.01$
