# Supplementary material for: Parenthood and care in academia: Publication counts, self-assessed productivity, and gendered differences
Source: PLoS One. 2026 Sep 16;21(9):e0355683. doi: 10.1371/journal.pone.0355683 (PMC13580985; doi:10.1371/journal.pone.0355683)
Supplement: Supplementary file 3 [file pone.0355683.s008.pdf]

Table S3: Effort Task and Parenthood

	(1)	(2)	(3)	(4)	(5)	(6)	(7)	(8)
Dummy Children								
No children	Ref.	Ref.	Ref.	Ref.	Ref.	Ref.	Ref.	Ref.
Yes children	5.312	5.082	3.702	0.481	0.650	0.771	0.744	-2.326
	(3.284)	(3.243)	(3.058)	(1.208)	(1.232)	(1.289)	(1.467)	(2.390)
Gender								
Man		Ref.	Ref.	Ref.	Ref.	Ref.	Ref.	Ref.
Woman		-1.417	-0.953	0.767	1.070	1.057	1.317	1.311
		(2.359)	(2.709)	(1.928)	(2.083)	(2.073)	(2.231)	(2.282)
Age			-0.865	-1.327	-1.365	-1.420	-1.336	-1.528
			(1.101)	(1.021)	(1.051)	(1.022)	(1.029)	(1.127)
Age sq.			0.013	0.019	0.020	0.021	0.020	0.023
			(0.015)	(0.014)	(0.015)	(0.014)	(0.015)	(0.016)
Desired N. of Children								
Does not want children				Ref.	Ref.	Ref.	Ref.	Ref.
1 child				0.422	-0.090	-0.041	-0.289	-1.098
				(1.016)	(1.095)	(1.085)	(1.221)	(1.842)
2 children				2.533	2.800	2.859	2.955	3.128
				(1.715)	(1.889)	(1.933)	(2.038)	(2.135)
3 or more children				-0.316	-0.343	-0.322	-0.118	-0.301
				(1.372)	(1.458)	(1.443)	(1.401)	(1.517)
Dummy Remote Work								
No					Ref.	Ref.	Ref.	Ref.
Yes					-1.834	-1.835	-1.794	-2.098
					(1.202)	(1.191)	(1.251)	(1.376)
Dummy Type of Employment								
Full-time						Ref.	Ref.	Ref.
Part-time						-0.997	-1.229	-0.963
						(1.672)	(2.001)	(1.941)
Income (scale)							-0.727	-0.707
							(0.830)	(0.807)
Weekly hours of childcare								0.124
								(0.130)
Diff. in childcare hours								-0.053
								(0.069)
Diff. in childcare hours sq.								-0.001
								(0.001)
Constant	17.347***	18.222***	32.084*	37.398**	39.277**	40.589**	41.693**	45.251**
	(0.277)	(1.421)	(18.301)	(16.997)	(17.498)	(16.507)	(16.733)	(18.519)
Observations	282	278	278	267	244	244	238	236
R^2	0.017	0.018	0.029	0.075	0.078	0.079	0.091	0.103

Standard errors in parentheses * $p < 0.10$, ** $p < 0.05$, *** $p < 0.01$
