# Supplementary material for: Parenthood and care in academia: Publication counts, self-assessed productivity, and gendered differences
Source: PLoS One. 2026 Sep 16;21(9):e0355683. doi: 10.1371/journal.pone.0355683 (PMC13580985; doi:10.1371/journal.pone.0355683)
Supplement: Supplementary file 5 [file pone.0355683.s010.pdf]

Table S5: Robustness checks

	Full model specifications								
	No outliers		*Logit*		*Probit*		*Control for effort*	*Control for nation*	
**Variable**	**Publications**	**Task**	**Publications**	**Task**	**Publications**	**Task**	**Publications**	**Publications**	**Task**
Dummy children	0.287	-0.413	0.997	-0.239	0.674	-0.149	0.290	0.258	-1.588
	(0.207)	(0.838)	(0.900)	(0.514)	(0.468)	(0.322)	(0.245)	(0.250)	(2.301)
Number of children	0.001	0.047	-0.136	-0.117	-0.051	-0.071	-0.005	-0.005	0.201
	(0.088)	(0.620)	(0.332)	(0.248)	(0.190)	(0.154)	(0.101)	(0.103)	(1.170)
Controls	✓	✓	✓	✓	✓	✓	✓	✓	✓
Observations	209	234	212	236	212	236	204	212	236

Standard errors in parentheses. * $p < 0.10$, ** $p < 0.05$, *** $p < 0.01$.
