# Supplementary material for: Parenthood and care in academia: Publication counts, self-assessed productivity, and gendered differences
Source: PLoS One. 2026 Sep 16;21(9):e0355683. doi: 10.1371/journal.pone.0355683 (PMC13580985; doi:10.1371/journal.pone.0355683)
Supplement: Supplementary file 6 [file pone.0355683.s011.pdf]

Table S6: Gender Interactions

	(1) Publications per year	(2) Productivity
Dummy Children		
No children	Ref.	Ref.
Yes children	0.407	-5.725
	(0.320)	(4.872)
Gender		
Man	Ref.	Ref.
Woman	-0.004	-0.970
	(0.241)	(0.891)
Yes children × Woman	-0.265	7.308
	(0.325)	(6.079)
Weekly hours of childcare	0.001	0.113
	(0.004)	(0.121)
Diff. in childcare hours	-0.005	-0.093
	(0.004)	(0.100)
Diff. in childcare hours sq.	-0.000**	-0.001
	(0.000)	(0.001)
Constant	-0.352	48.866**
	(1.317)	(20.050)
Observations	212	236
R^2	0.168	0.115

Standard errors in parentheses * $p < 0.10$, ** $p < 0.05$, *** $p < 0.01$
