# Supplementary material for: Parenthood and care in academia: Publication counts, self-assessed productivity, and gendered differences
Source: PLoS One. 2026 Sep 16;21(9):e0355683. doi: 10.1371/journal.pone.0355683 (PMC13580985; doi:10.1371/journal.pone.0355683)
Supplement: Supplementary file 7 [file pone.0355683.s012.pdf]

Table S7: Interaction Gender Diff. Hours of Care

	(1) Publications per year	(2) Productivity
Dummy Children		
No children	Ref.	Ref.
Yes children	0.253	-2.158
	(0.235)	(2.300)
Gender		
Man	Ref.	Ref.
Woman	-0.120	1.425
	(0.170)	(2.361)
Man × Diff_Hours_care	0.000	0.000
Woman × Diff_Hours_care	0.012	-0.097
	(0.008)	(0.093)
Weekly hours of care	0.001	0.125
	(0.004)	(0.130)
Diff_Hours_care	-0.012*	-0.008
	(0.006)	(0.052)
Diff Hours care square	-0.000***	-0.000
	(0.000)	(0.001)
Controls	✓	✓
Constant	-0.166	44.780**
	(1.215)	(18.359)
Observations	212	236
R^2	0.171	0.105

Standard errors in parentheses
* $p < 0.10$, ** $p < 0.05$, *** $p < 0.01$
