# Supplementary material for: Parenthood and care in academia: Publication counts, self-assessed productivity, and gendered differences
Source: PLoS One. 2026 Sep 16;21(9):e0355683. doi: 10.1371/journal.pone.0355683 (PMC13580985; doi:10.1371/journal.pone.0355683)
Supplement: Supplementary file 8 [file pone.0355683.s013.pdf]

Table S8: Parenthood and Productivity: Panel Data FE Model

	(1) Overall	(2) Men	(3) Women
Had a child	0.496**	0.556*	-0.328
	(0.194)	(0.304)	(0.351)
Age	0.268***	0.457***	-0.088
	(0.044)	(0.102)	(0.216)
Age sq.	-0.001***	-0.003***	0.002
	(0.001)	(0.001)	(0.003)
Constant	-5.576***	-8.936***	2.420
	(0.895)	(2.195)	(4.357)
Observations	924	522	193
R^2	0.214	0.172	0.099

Standard errors in parentheses

* $p < 0.10$, ** $p < 0.05$, *** $p < 0.01$
