# Supplementary material for: Parenthood and care in academia: Publication counts, self-assessed productivity, and gendered differences
Source: PLoS One. 2026 Sep 16;21(9):e0355683. doi: 10.1371/journal.pone.0355683 (PMC13580985; doi:10.1371/journal.pone.0355683)
Supplement: Supplementary file 9 [file pone.0355683.s014.pdf]

Table S9: Parenthood and Productivity: Panel Data FE Model

	(1) Overall	(2) Men	(3) Women
Had a child	0.761	0.501	0.030
	(0.501)	(0.767)	(0.758)
Had a child (t-1)	-0.100	0.009	-0.258
	(0.484)	(0.716)	(0.675)
Age	0.276***	0.950***	-0.144
	(0.047)	(0.190)	(0.288)
Age sq.	-0.002***	-0.009***	0.003
	(0.001)	(0.002)	(0.003)
Constant	-5.902***	-20.074***	3.635
	(0.967)	(4.168)	(5.986)
Observations	868	485	174
R^2	0.220	0.181	0.085

Standard errors in parentheses

* $p < 0.10$, ** $p < 0.05$, *** $p < 0.01$
