# Supplementary material for: Parenthood and care in academia: Publication counts, self-assessed productivity, and gendered differences
Source: PLoS One. 2026 Sep 16;21(9):e0355683. doi: 10.1371/journal.pone.0355683 (PMC13580985; doi:10.1371/journal.pone.0355683)
Supplement: Supplementary file 10 [file pone.0355683.s015.pdf]

Table S10: Parenthood and Publication Quality: Panel Data FE Models

	**H-index**			**Journal Quartile**			**Journal Rank**		
	Overall	Men	Women	Overall	Men	Women	Overall	Men	Women
Had a child	1.309	4.662	-16.791	0.054	0.058	0.055	-93.867	-206.201	625.092
	(7.867)	(8.743)	(18.725)	(0.081)	(0.086)	(0.224)	(475.844)	(484.562)	(1384.694)
Age	4.734*	3.859	16.331	0.023	0.025	-0.043	91.413	138.297	-754.571
	(2.784)	(2.948)	(11.538)	(0.029)	(0.029)	(0.138)	(168.395)	(163.366)	(853.273)
Age sq.	-0.035	-0.027	-0.168	-0.000	-0.000	0.001	-0.194	-0.708	10.018
	(0.031)	(0.032)	(0.139)	(0.000)	(0.000)	(0.002)	(1.882)	(1.800)	(10.286)
Constant	-72.236	-55.410	-303.975	2.059***	1.997***	3.417	124.278	-1103.316	17613.982
	(59.323)	(63.140)	(232.299)	(0.614)	(0.618)	(2.780)	(3588.117)	(3499.337)	(17178.508)
Observations	715	522	193	715	522	193	715	522	193
R^2	0.019	0.021	0.025	0.007	0.008	0.007	0.008	0.010	0.010

Standard errors in parentheses. * $p < 0.10$, ** $p < 0.05$, *** $p < 0.01$.
