# Supplementary material for: Parenthood and care in academia: Publication counts, self-assessed productivity, and gendered differences
Source: PLoS One. 2026 Sep 16;21(9):e0355683. doi: 10.1371/journal.pone.0355683 (PMC13580985; doi:10.1371/journal.pone.0355683)
Supplement: Supplementary file 11 [file pone.0355683.s016.pdf]

Table S11: Self-reported productivity loss from parenthood

	(1)
Woman	-0.705
	(0.601)
Diff. in childcare hrs	0.001
	(0.010)
Diff. in childcare hrs sq.	0.000
	(0.000)
Woman × Diff. in childcare hrs	0.048**
	(0.019)
Woman × Diff. in childcare hrs hrs sq. hrs	0.000
	(0.000)
Controls	✓
Constant	-3.870
	(3.590)
Observations	93
R^2	0.392

Standard errors in parentheses

* $p < 0.10$, ** $p < 0.05$, *** $p < 0.01$
