# Supplementary material for: Parenthood and care in academia: Publication counts, self-assessed productivity, and gendered differences
Source: PLoS One. 2026 Sep 16;21(9):e0355683. doi: 10.1371/journal.pone.0355683 (PMC13580985; doi:10.1371/journal.pone.0355683)
Supplement: Supplementary file 12 [file pone.0355683.s017.pdf]

Table S12: Perceptions of Parenthood (Multinomial Logit)

	(1) Parenthood as advantage	(2) Self-perception as worker	(3) Colleagues' perception as worker
**Outcome: No (vs. I don't know)**			
Woman	2.298**	3.538**	0.471
	(0.929)	(1.431)	(0.576)
Age	0.342	-0.513*	-0.410**
	(0.227)	(0.271)	(0.206)
Age sq.	-0.004	0.005*	0.005*
	(0.003)	(0.003)	(0.002)
Ideal number of children	1.115**	0.525	0.083
	(0.569)	(0.532)	(0.400)
Remote work (yes)	2.189*	-14.211***	0.173
	(1.128)	(1.196)	(0.945)
Part-time	0.564	-2.341	0.336
	(0.964)	(1.424)	(0.721)
Income (scale)	-0.198	-0.415	0.108
	(0.233)	(0.408)	(0.212)
Weekly childcare hours	-0.017	-0.001	-0.005
	(0.016)	(0.028)	(0.013)
Care hours imbalance	-0.011	-0.010	-0.014
	(0.015)	(0.021)	(0.014)
Care imbalance squared	0.000	-0.001**	0.000
	(0.000)	(0.000)	(0.000)
Constant	-10.349*	27.994***	7.565
	(5.348)	(6.634)	(4.679)
**Outcome: Yes (vs. I don't know)**			
Woman	0.890	2.767**	0.281
	(0.902)	(1.295)	(0.699)
Age	0.000	-0.246	-0.349*
	(0.227)	(0.234)	(0.208)
Age sq.	0.000	0.002	0.004*
	(0.003)	(0.003)	(0.002)
Ideal number of children	1.378**	0.732	0.528
	(0.599)	(0.513)	(0.559)
Remote work (yes)	0.990	-12.719***	0.885
	(1.014)	(1.053)	(1.074)
Part-time	1.484*	-0.768	-0.082
	(0.824)	(0.882)	(0.736)
Income (scale)	-0.153	-0.019	-0.333
	(0.248)	(0.314)	(0.238)
Weekly childcare hours	-0.010	-0.005	-0.041**
	(0.013)	(0.019)	(0.020)
Care hours imbalance	0.003	-0.001	0.005
	(0.017)	(0.017)	(0.016)
Care imbalance squared	-0.000	-0.001**	0.000
	(0.000)	(0.000)	(0.000)
Constant	-2.513	19.312***	8.057
	(5.097)	(5.970)	(4.998)
Observations	93	92	93

Notes: Multinomial logit coefficients (log-odds). The reference category is *I don't know*.
Robust standard errors in parentheses. * $p < 0.10$, ** $p < 0.05$, *** $p < 0.01$.
