# Supplementary material for: Parenthood and care in academia: Publication counts, self-assessed productivity, and gendered differences
Source: PLoS One. 2026 Sep 16;21(9):e0355683. doi: 10.1371/journal.pone.0355683 (PMC13580985; doi:10.1371/journal.pone.0355683)
Supplement: Supplementary file 13 [file pone.0355683.s018.pdf]

Table S13: Excluded studies

Title	Authors	Year	Reason
Balancing science and family: Tidbits of wisdom from those who've tried it and succeeded	Bascom-Slack C. A.	2011	Not relevant to the review objective
Penalties and premiums: The impact of gender, marriage, and parenthood on faculty salaries in science, engineering and mathematics (SEM) and non-SEM fields	Kelly K. et al.	2012	Ineligible population/intervention/outcome
Gender Norms and Institutional Culture: The Family-Friendly Versus the Father-Friendly University	Sallee, MW	2013	Not relevant to the review objective
Parenthood and Productivity of Highly Skilled Labor: Evidence from the Groves of Academe	Krapf M. et al.	2014	Ineligible population/intervention/outcome
Retaining female postgraduates in academia: the role of gender and prospective parenthood	Crabb S. et al.	2014	Ineligible population/intervention/outcome
Strategies of academic parents to manage work-life conflict in research abroad	Lubitow A. et al.	2014	Not relevant to the review objective
Carving a "Third Path": Faculty Parents' Resistance to the Ideal Academic Worker Norm	Ollilainen M. et al.	2014	Ineligible population/intervention/outcome
Retaining female postdocs in academia	van der Weijden K. et al.	2015	Ineligible population/intervention/outcome
Gender family and academic careers in Turkey	Inanc H. et al.	2016	Ineligible population/intervention/outcome
Images, ideals and constraints in times of neoliberal transformations: Reproduction and profession as conflicting or complementary spheres in academia?	Bomert C. et al.	2017	Ineligible population/intervention/outcome
The parent track: Timing balance and choice in academia	DeRoche C. et al.	2017	Ineligible population/intervention/outcome
The success rate of female doctoral students with young children in balancing parenting and studying	Harvankova K.	2017	Ineligible population/intervention/outcome

Continues on the next page

Table S13: Excluded studies

Title	Authors	Year	Reason
Gender and Research Productivity: Do Parenthood and Academic Social Capital Explain Productivity Differences during the Doctoral Phase?	Jaksztat S.	2017	Ineligible population/intervention/outcome
Understanding gender inequality and the role of the work/family interface in contemporary academia: An introduction	Dubois-Shaik F. et al.	2017	Not relevant to the review objective
Gender representation in Russian academic journals	Krasnyak O.	2017	Not relevant to the review objective
Academic couples, parenthood and women's research careers	Vohlídalová, M	2017	Not relevant to the review objective
Pregnancy, motherhood, and academic career goals: Doctoral students' perspectives	Mirick R. G. et al.	2018	Not relevant to the review objective
Strategies for balance: Examining how parents of color navigate work and life in the academy	Novich M. et al.	2018	Not relevant to the review objective
Renewable Energy in Urban Areas: Worldwide Research Trends	Perea-Moreno, MA; Hernandez-Escobedo, Q; Perea-Moreno, AJ	2018	Not relevant to the review objective
Challenges facing parents in academia	Weeks K. L.	2018	Not relevant to the review objective
Playing in the corridors of academia	Nislev E. et al.	2018	Not relevant to the review objective
Models of parenting and its effect on academic productivity: Preliminary results from an international survey	Derrick G. E. et al.	2019	Ineligible population/intervention/outcome
Academic careers and parenting: identity, performance and surveillance	Harris C. et al.	2019	Ineligible population/intervention/outcome
Academic mothers as ideal workers in the USA and Finland	Ollilainen M.	2019	Not relevant to the review objective
Motherhood and academic careers in Indonesia	Toyibah D.	2019	Not relevant to the review objective

Continues on the next page

Table S13: Excluded studies

Title	Authors	Year	Reason
Parenting as a Moderating Factor for Research Productivity and Work-Life Balance: Evidence from Philippine Women Academics	Dapiton E. P. et al.	2020	Not relevant to the review objective
Parenting Through Academia as a SICB member	Schwaner M. J. et al.	2020	Ineligible population/intervention/outcome
Academic Productivity Differences by Gender and Child Age in Science, Technology, Engineering, Mathematics, and Medicine Faculty During the COVID-19 Pandemic	Krukowski R. A. et al.	2021	Ineligible population/intervention/outcome
Evolving Trends and Research Hotspots in Disaster Epidemiology From 1985 to 2020: A Bibliometric Analysis	Liu, T; Liu, X; Li, Y; Liu, SY; Cao, CX	2021	Not relevant to the review objective
Motherhood and academic careers: Challenges faced by African American women in academia marriage parenthood and the tenure clock	Jackson-Nevels B.	2021	Ineligible population/intervention/outcome
Gender Race and Parenthood Impact Academic Productivity During the COVID-19 Pandemic: From Survey to Action	Staniscuaski F. et al.	2021	Ineligible population/intervention/outcome
Conforming, accommodating, or resisting? How parents in academia negotiate their professional identity	Van Engen M. L. et al.	2021	Not relevant to the review objective
Less money, less children, and less prestige: Differences between male and female academic librarians	Eva N. et al.	2021	Not relevant to the review objective
The relationship between parenting engagement and academic performance	Derrick G. E. et al.	2022	Ineligible population/intervention/outcome
Gender, Parenting Status, and the Academic Productivity of Pediatricians During the COVID-19 Pandemic	Sharp E. A. et al.	2022	Ineligible population/intervention/outcome

Continues on the next page

Table S13: Excluded studies

Title	Authors	Year	Reason
Back to zero? Precarious employment in academia amongst 'older' early career researchers a life-course approach	Spina N. et al.	2022	Ineligible population/intervention/outcome
A dichotomy of domestic and academic pathways: Challenges of motherhood in an international doctoral program on land science	Velander S. et al.	2022	Not relevant to the review objective
Academic mothers with disabilities: Navigating academia and parenthood during COVID-19	Wagner K. et al.	2022	Not relevant to the review objective
How parenthood contributes to gender gaps in academia	Zheng X. et al.	2022	Ineligible population/intervention/outcome
Parenthood and Gender in Russian Academia	Chechik E. A.	2023	Not relevant to the review objective
Effects of parenthood and gender on well-being and work productivity among Canadian academic research faculty amidst the COVID-19 pandemic.	Gordon J. L. et al.	2023	Ineligible population/intervention/outcome
Female advantage in German sociology: Does accounting for the "leaky pipeline" effect in becoming a tenured university professor make a difference?	Habicht I. M. et al.	2023	Ineligible population/intervention/outcome
The influence of departmental cultuer on academic parents' pro-work behaviors	Kmec J. A. et al.	2023	Not relevant to the review objective
Negotiating dance academia and parenting: a conversation between colleagues and friends	Milovanović D. et al.	2023	Not relevant to the review objective
Gendered dynamics of academic networks	Scalera Elliott J. et al.	2023	Not relevant to the review objective
Gender and retention patterns among U.S. faculty	Spoon K. et al.	2023	Not relevant to the review objective
Bias against parents in science hits women harder	Staniscuaski F. et al.	2023	Not relevant to the review objective
Father involvement and gender equality in the United States: contemporary norms and barriers	Boeri, N	2024	Not relevant to the review objective

Continues on the next page

Table S13: Excluded studies

Title	Authors	Year	Reason
Balancing work, life, and care responsibilities in academia: the male perspective	Cannito M. et al.	2024	Not relevant to the review objective
Motherhood in academia: A novel dataset of UK academic women with an application to maternity leave uptake	Di Leo R. et al.	2024	Ineligible population/intervention/outcome
Motherhood in the Music Academy	Fitzpatrick K. et al.	2024	Not relevant to the review objective
Muted voices of invisible men: the impact of male childlessness	Hadley R. A.	2024	Not relevant to the review objective
COVID-19 as a chronic stressor and the importance of individual identity: A data-driven look at academic productivity during the pandemic	Harris B. N. et al.	2024	Not relevant to the review objective
The gendered effects of parenthood on career outcomes: Do family gaps explain gender differences along the science pipeline?	Lopes M.	2024	Ineligible population/intervention/outcome
Getting ahead in the social sciences: How parenthood and publishing contribute to gender gaps in academic career advancement	Nielsen M. W. et al.	2024	Ineligible population/intervention/outcome
How does parenthood affect an ICT practitioner's work? A survey study with fathers	Rocha L. et al.	2024	Ineligible population/intervention/outcome
UNIVERSITY MATURITY MODEL-A BIBLIOMETRIC ANALYSIS	Szydlo, J; Sakowicz, A; di Pietro, F	2024	Not relevant to the review objective
Creating good knowledge together: heartful-communal storytelling at the intersection of caregiving and academia	Winham N. et al.	2024	Not relevant to the review objective
Agency and cruel optimism in the care obfuscations of UK-based academic mothers	Etheridge M.	2025	Not relevant to the review objective
Evidence of a parent premium in the classroom: positive perceptions of professors with children	Nazione S. et al.	2025	Not relevant to the review objective

Continues on the next page

Table S13: Excluded studies

Title	Authors	Year	Reason
Mapping Orthopedic Research Trends in Nepal (2000-2024): A Scopus-Based Bibliometric Study	Regmi, A; Niraula, BB	2025	Not relevant to the review objective
Gendered Challenges in Academia: Exploring the Impact of Working Hours Stress and Job Satisfaction Among Mid-Level University Staff in Germany	Röbken H. et al.	2025	Not relevant to the review objective
This is us now. Collectively making sense of our new identities as parents and early career academics	Scholz F. et al.	2025	Ineligible population/intervention/outcome
Supporting academic parents: The effects of dependent care policies on research productivity trends	Van Egdom D. et al.	2025	Ineligible population/intervention/outcome
Exploring passengers' preferences for high-speed rails: A CiteSpace bibliometric analysis	Wu, D; Martín, JC	2025	Not relevant to the review objective
Graduate students' mental health during COVID-19. Examining the Cognitive and Psychological Effects of the COVID-19 Global Pandemic on High School, College, and Graduate Learners	Yoo H. J. et al.	2025	Not relevant to the review objective
Invisible Sportswomen 4.0—"Everyone Will Know You're a Mum It's Part of Your Resume": A Mixed-Methods Cross-Sectional Study Exploring the Impact of Family on Women's Experiences in Exercise and Sport Science Academia	Marks K. et al.	2025	Ineligible population/intervention/outcome
The gender pay gap at the early stages of academic careers	Magda I. et al.	2025	Not relevant to the review objective
